# Enhancing Head and Neck Tumor Segmentation in MRI: The Impact of Image Preprocessing and Model Ensembling

**DOI:** 10.1007/978-3-031-83274-1_8

**Published:** 2025-03-03

**Authors:** Mehdi Astaraki, Iuliana Toma-Dasu

**Affiliations:** 1 Department of Medical Radiation Physics, Stockholm University, Stockholm, Sweden; 2 Department of Oncology-Pathology, Karolinska Institutet, Solna, Sweden

**Keywords:** head-neck tumor, MR-guided radiotherapy, GTV, segmentation

## Abstract

The adoption of online adaptive MR-guided radiotherapy (MRgRT) for Head and Neck Cancer (HNC) treatment faces challenges due to the complexity of manual HNC tumor delineation. This study focused on the problem of HNC tumor segmentation and investigated the effects of different preprocessing techniques, robust segmentation models, and ensembling steps on segmentation accuracy to propose an optimal solution. We contributed to the MICCAI Head and Neck Tumor Segmentation for MR-Guided Applications (HNTS-MRG) challenge which contains segmentation of HNC tumors in Task1) pre-RT and Task2) mid-RT MR images. In the internal validation phase, the most accurate results were achieved by ensembling two models trained on maximally cropped and contrast-enhanced images which yielded average volumetric Dice scores of (0.680, 0.785) and (0.493, 0.810) for (GTVp, GTVn) on pre-RT and mid-RT volumes. For the final testing phase, the models were submitted under the team’s name of “Stockholm_Trio” and the overall segmentation performance achieved aggregated Dice scores of (0.795, 0.849) and (0.553, 0.865) for pre- and mid-RT tasks, respectively. The developed models are available at https://github.com/Astarakee/miccai24

## 1 Introduction

Head and neck cancer (HNC) encompasses a diverse range of malignancies arising within the anatomical structures of the head and neck, including the oral cavity, nasopharynx, oropharynx, larynx, and hypopharynx [1, 2].

Approximately half of all cancer patients undergo radiation therapy (RT). Successful treatment hinges on the accurate and precise delivery of radiation to the targeted areas while minimizing damage to adjacent healthy tissue. Intensity-modulated radiation therapy as the most common treatment modality for HNC enabled conformal dose delivery through technological advancements. Magnetic Resonance (MR) imaging linear accelerators combine these two technologies to provide improved soft tissue contrast in real-time. MR-guided RT (MRgRT) has a number of advantages over conventional RT, including superior soft tissue contrast compared to CT, continuous intrafraction MR imaging, and the ability to adapt treatment plans in real-time. Online adaptive MRgRT, performed while the patient remains in the treatment position, takes into account real-time anatomical changes like soft-tissue deformity and volume changes. Clinically, these technical advantages result in the ability to safely escalate the dose while reducing toxicity and treatment time. As a result, the use of MRgRT techniques has increased over time, particularly in the treatment of pancreatic, prostate, lung, and liver cancers [3, 4].

HNC tumors present significant challenges for manual delineation due to their complex anatomical structures. Furthermore, the sheer volume of data acquired for MRgRT planning makes manual segmentation almost impractical. Thus, developing accurate and automated segmentation methods for HNC tumors is crucial for streamlining the delineation process and ensuring greater consistency in contouring. The advancements in Deep Learning (DL) techniques over the past decade have resulted in the development of sophisticated DL models that demonstrate exceptional performance in various medical imaging applications, notably tumor segmentation [5–8]. Within the context of HNC segmentation, in recent years, three challenges have been established to benchmark algorithmic progress. The Head and Neck Tumor (HECKTOR) challenge [9] focused on evaluating the segmentation of primary and nodal gross tumors in PET-CT images. In this challenge, top performing models achieved Dice scores in the range of 0.77, through the utilization of UNet-based models enhanced with a variety of techniques. These techniques included the incorporation of attention mechanisms, residual connections, image preprocessing, and model ensembling strategies. The segmentation of OARs and GTVs of nasopharyngeal carcinoma for radiotherapy planning (SegRap) challenge [10] assessed the segmentation of both tumors and 45 healthy structures in bi-modal CT images. Top performing models in the GTVs segmentation task utilized preprocessing techniques, notably intensity harmonization and background cropping, prior to model training. Encoder-decoder segmentation models including nnU-Net [6] and its modified version, as well as MultiTalent [7] resulted in average Dice scores reaching 0.73. Lastly, the Head and Neck Segmentation (HaN-Seg) challenge [10] aimed to benchmark the performance of automatic segmentation across 30 OARs in multimodal CT-MRIs. Nevertheless, the objective assessments of MR-only tumor segmentation techniques for MRgRT applications have not been investigated thoroughly.

The MICCAI Head and Neck Tumor Segmentation for MR-Guided Applications (HNTS-MRG) 2024 challenge [11] has been focused on the segmentation of HNC tumors for MRI-guided Adaptive RT applications. This paper presents our contribution to this challenge in which we conducted a set of extensive experiments to identify the best workflow for HNC tumor segmentation in MR images.

## 2 Materials and Methods

The HNTS-MRG challenge focuses on the automated segmentation of gross tumor volumes (GTVs) from images acquired at two distinct time points in two independent tasks: task 1) pre-RT MR images and task 2) mid-RT MR images. The core objective of this challenge is to investigate whether integrating prior timepoint data into the algorithms can improve the segmentation accuracy of primary GTV (GTVp) and metastatic lymph node GTV (GTVn).

### 2.1 Studied Datasets

All data utilized in this study were collected from patients with histologically confirmed head and neck cancer (HNC), primarily oropharyngeal cancer, who underwent radiotherapy (RT) at The University of Texas MD Anderson Cancer Center. Imaging data included a mixed set of fat-suppressed and non-fat-suppressed T2-weighted (T2w) sequences of the head and neck region.

Both pre- and mid-RT volumes were examined by three to four independent expert physicians, and manual delineation was conducted by reviewing the patient’s medical history, including relevant previous imaging such as PET-CT. The obtained segmentation masks were then reviewed by an experienced radiation oncologist before utilizing the STAPLE algorithm to generate the final segmentation masks.

The training dataset comprises 150 T2w MRIs accompanied by their corresponding segmentation masks. The developed algorithms are mandated to be containerized and submitted to an online platform for an objective assessment on an unseen testing set encompassing 50 subjects.

#### Task 1: Pre-RT

The pre-RT images were acquired one to three weeks prior to the commencement of RT. During the training phase of model development, participants had the option to utilize exclusively pre-RT volumes or to incorporate mid-RT volumes to expand their training dataset. However, the testing phase exclusively involved pre-RT volumes.

#### Task 2: Mid-RT

The mid-RT volumes were acquired two to four weeks after the initiation of RT. In addition to the mid-RT volumes, for each subject, the pre-RT volumes were included in two distinct geometrical configurations: the original volume and the ones deformably registered to the mid-RT volumes. Essentially, the pre-RT data could function as prior knowledge, and participants had the flexibility to employ any desired combination settings between the pre- and mid-RT volumes.

**Fig. 1.**
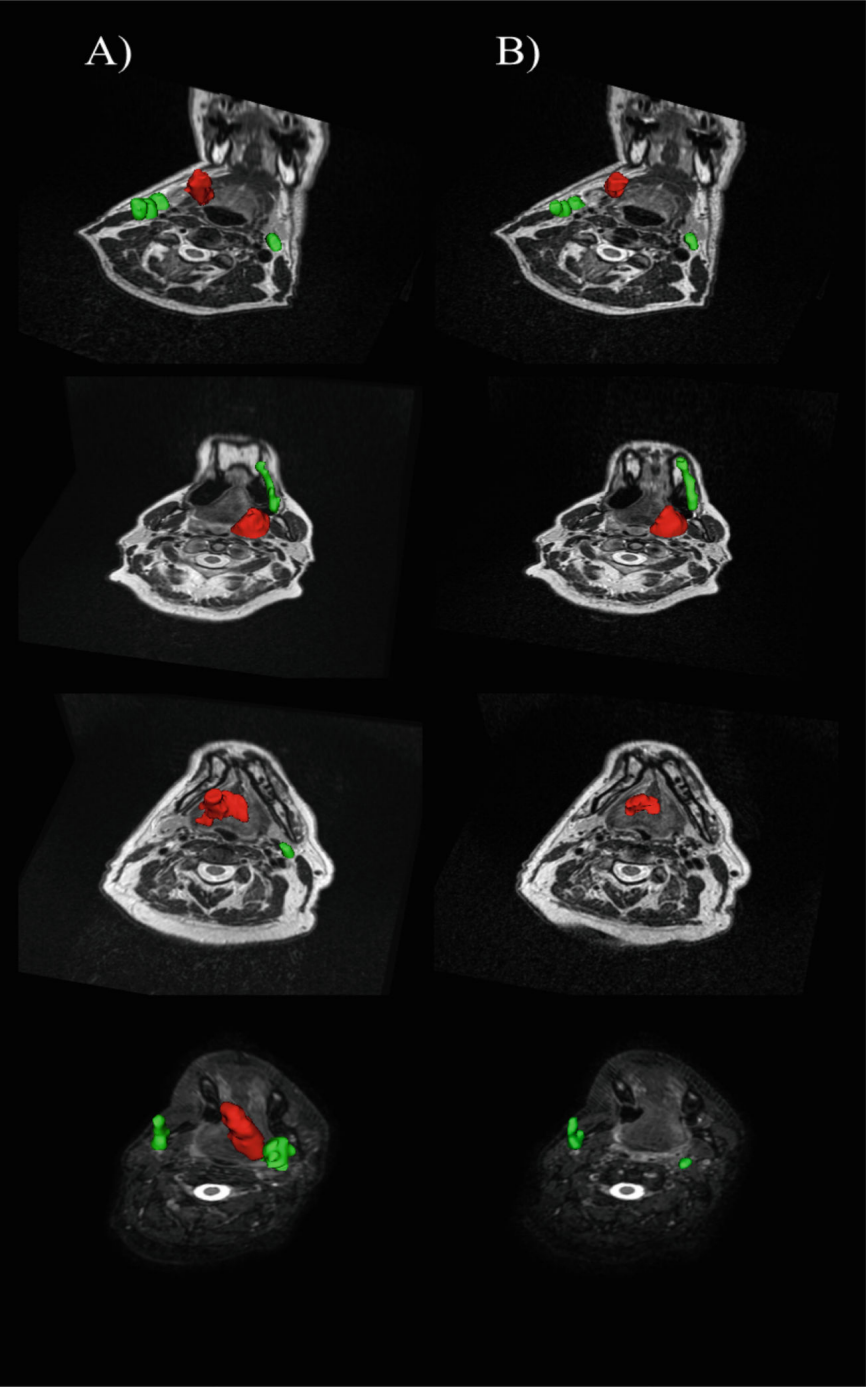
Illustrative examples of tumor appearance in pre-RT (left column) and mid-RT (right column) images. Primary tumors are depicted in red and metastatic lymph nodes are visualized in green. Pseudo-3D visualization in axial-coronal views was employed to provide a comprehensive context of tumor volumes and their relative positions.

Figure 1 illustrates examples of the tumor appearance in the examined training subjects.

### 2.2 Methods

#### Preprocessing

The provided image volumes by the organizer were already cropped to encompass the region from the superior aspect of the clavicles to the inferior aspect of the nasal septum. This standardized the field of view and removed identifiable facial features.

To further refine the datasets for model training, a two-stage preprocessing pipeline was implemented. In the first stage, to optimize patch extraction and minimize background inclusion, MR volumes were cropped to maximally exclude background regions. Thresholding and connected component analysis were employed to segment the volumes into foreground (body) and background, utilizing the body skin as the delineating boundary. A bounding box was then generated to enclose the widest portion of the volume, ensuring optimal loading of head and neck tissues within each patch while eliminating unnecessary background information. The second preprocessing stage focused on intensity enhancement. The N4 bias correction field algorithm [12] was applied to mitigate intensity inhomogeneities within each MR volume. Subsequently, to address the substantial variability in the dynamic range of MR volume intensities, cases with peak intensities exceeding 700 underwent clipping of values above the 99th percentile. Finally, Z-score standardization was performed as the concluding intensity modification step.

#### SegResNet Model

The encoder-decoder network architecture of MONAI SegResNet [13] contains ResNet blocks in the encoder part where each block consists of two convolution layers, with normalization and skip connections. The decoder part consists of a single block per spatial level. Each block within the decoder path starts by reducing the depth of feature maps, doubling the spatial dimension, followed by skipping connections. It should be noted that the employed models did not include the variational decoder branch. The models were trained for 500 epochs with the following hyperparameters: variable training and validation iterations per epoch to cover all the samples, batch size of 4, initial learning rate of 1e−4, LeakyReLU as activation function, instance normalization, four levels of deep supervision, and patch sizes were (96 × 160 × 128). The network encoder consisted of six stages with (1, 3, 4, 4, 6, 6) number of convolutions.

#### nnU-Net ResENC Model

The nnU-Net model V2 [6] served as the second segmentation pipeline. A ResNet-enhanced U-Net (“nnU-Net ResENCM”) with original configurations [14] was employed. Models were trained for 1200 epochs across five folds with 250 training and 50 validation iterations, an initial learning rate of 1e-2, a batch size of 4, enabled deep supervision, and a patch size of (64 × 224 × 160). The network architecture include six encoder stages with (32, 64, 128, 256, 320, 320) kernels per stage.

#### MedNeXt Model

MedNeXt [15] is a network architecture composed exclusively of ConvNeXt blocks [16] thereby fully leveraging the latter’s design advantages. To preserve contextual information during upsampling and downsampling operations, the architecture replaces standard up-down/sample blocks with Residual Inverted Bottlenecks. To mitigate performance saturation associated with large kernel sizes, training commences with smaller kernels but can be progressively increased through the UpKern technique. In general, MedNeXt is a scalable encoder-decoder framework optimized for 3D segmentation tasks, designed to maximize the potential of ConvNeXt architectures within the constraints of limited datasets. The MedNeXt model was compiled using large-scale network architectures with a kernel size of 3, a batch size of 2, isotropic spacing of 1mm, and a patch size of (128 × 128 × 128), following the nnU-Net V1 training protocol.

#### U-Mamba Model

Recent advancements in structured state-space sequence modeling, exemplified by the S4 architecture [17], have established these models as efficient building blocks for complex deep networks. Building upon this foundation, Mamba [18] introduced a selective mechanism to enhance S4’s capability to focus on relevant input information. The U-Mamba [19] architecture further innovates by integrating state-space modules into convolutional blocks, enabling a hybrid approach that effectively captures both localized features and long-range dependencies within image data. This model offers a compelling alternative to transformer-based models by providing linear scaling with respect to feature size, in contrast to the quadratic complexity inherent in the self-attention mechanisms of transformers. In this study, we investigated the functionality of the U-Mamba model for both tasks by using the original implementation within the nnU-Net V2 framework.

#### Model Development and Ensembling

**Fig. 2.**
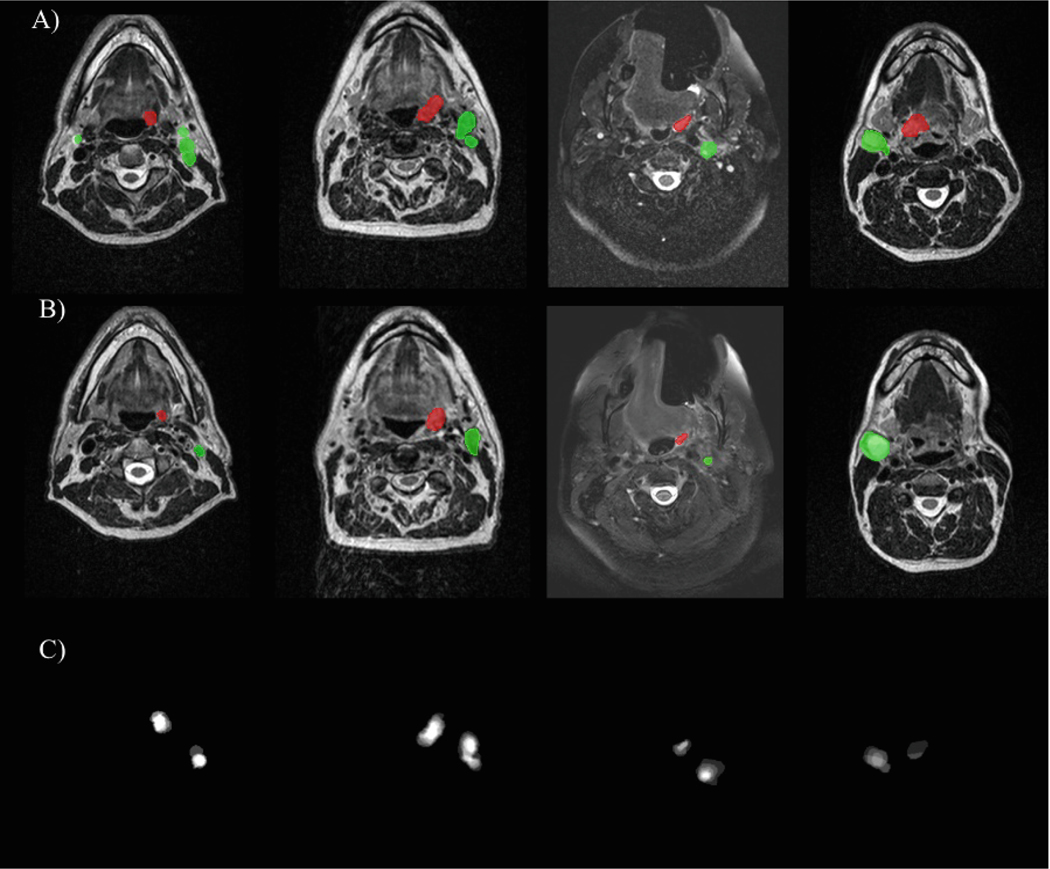
Examples of preDistance-prior calculated from segmentation masks of first time-point MRIs. Axial slices showcasing preRT (row A), mid-RT (row B), and prior channel (row C).

All the described models were optimized with a combination of Cross Entropy and Dice loss. For the pre-RT task, all models were trained exclusively on pre-RT MR volumes to segment the images into GTVp, GTVn, and background. In contrast, for the mid-RT task, an ablation study was conducted to explore different combinations of image data and segmentation masks. These settings included training the model using only mid-RT MR volumes (*no-prior*), incorporating registered pre-RT MRs as a second channel (*preMR-prior*), including pre-RT segmentation masks as the second channel (*preMask-prior*), and integrating registered pre-RT MR and segmentation masks together into a three-channel model (*preMRMask-prior*). Additionally, we dilated the pre-RT masks with 3 × 3 × 3 structure elements, then signed distance maps were derived from the dilated masks and incorporated as prior information to guide the network’s attention (*preDistance-prior*). Figure 2 shows examples of distance maps generated from pre-RT masks.

To enhance the overall model’s robustness, the complementary roles of the developed models were assessed. To this end, the best-performing models for GTVp and GTVn were combined to generate the final segmentation masks.

## 3 Results

The segmentation models were trained with a 5-fold cross-validation resampling strategy on the training data set except for the MedNeXt model which was trained with 3-folds. For better readability, only the quantified Dice metrics are reported in this paper. Table 1 shows the segmentation results for pre-RT task over the training set.

Table 1 reveals that ResENC and ModNeXt models consistently surpassed other models in performance, particularly when trained on the preprocessed dataset. Consequently, these two models were chosen for the mid-RT ablation experiments. However, due to challenges in optimizing the MedNeXt model for this task, only the nnU-Net ResENC model was ultimately utilized for training. The results of the ablation experiments conducted with various combinations of image and mask data for the mid-RT task are presented in Table 2.

**Fig. 3.**
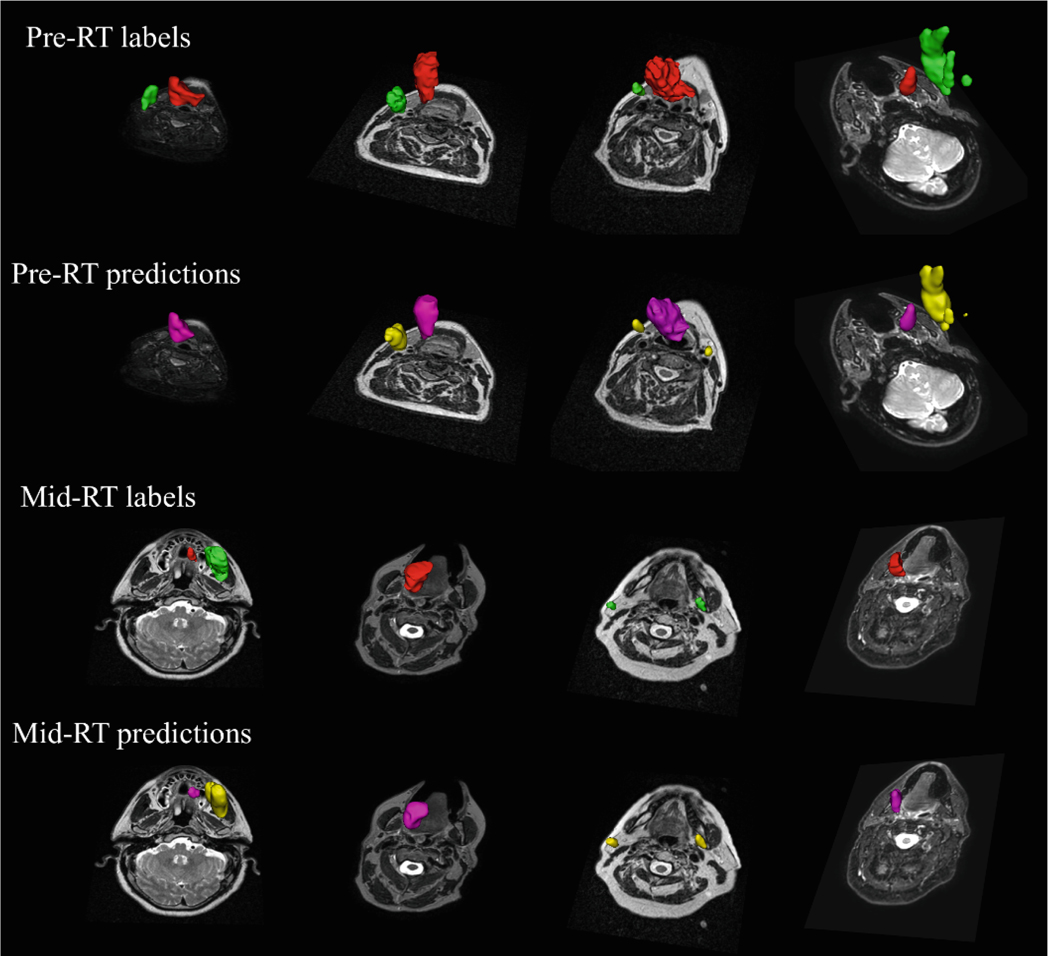
Displaying predicted segmentation masks for pre-RT and mid-RT tasks. For each task, the upper row presents ground truth masks with GTVp in red and GTVn in green. The lower row illustrates corresponding predicted masks, where GTVp is shown in magenta and GTVn in yellow. Pseudo-3D visualization in axial views was employed to provide a comprehensive context of tumor volumes and their relative positions.

The results displayed in Table 2 clearly indicate the superior performance of the *preDistance-prior* settings compared to other models. Figure 3 provides a visual representation of the segmentation performance achieved by the top-performing models for both pre-RT and mid-RT tasks.

In a nutshell, the empirical results obtained on the training dataset demonstrate that the implemented preprocessing techniques significantly enhanced the segmentation accuracy of the models. The optimal results were attained by ensembling ResENC and MedNeXt models for the pre-RT task and incorporating distance maps as prior knowledge for the mid-RT task. Notably, the inference time for the pre-RT task was under 6 min and approximately 3 min for the mid-RT task, utilizing a local workstation with limited resources (12GB GPU memory, 32GB RAM, and 8 CPU threads). However, the execution time of the Docker image on the evaluation platform surpassed the 20-min limit, resulting in failed job submissions. Consequently, to meet the resource limitations, the final submitted models underwent substantial simplifications. Specifically, the preprocessing pipeline was simplified by removing both bias correction and volume cropping. Furthermore, the ensemble approach initially planned for the pre-RT task, which involved both ResENC and MedNeXT models, was revised to utilize only three trained folds of the MedNeXT model. Similarly, for the mid-RT task, only three folds of the ResENC model were employed. The performance of these simplified submitted models on both the preliminary development phase and final testing datasets is presented in Table 3. Furthermore, the final ranking of the challenge was determined by calculating the average of aggregated Dice metrics (mean DSC_agg_) over the GTVp and the GTVn. The resulting mean DSC_agg_ values of this contribution were 0.822 and 0.710 for the pre-RT and mid-RT tasks, respectively.

## 4 Discussion

Accurate delineation of the GTVs is critical for effective RT planning in HNC. However, the complex morphology of head and neck structures and low target-to-background contrast present remarkable challenges for delineation tasks. In addition, manual delineation is time-consuming and prone to inter-observer variability, particularly in MRgRT applications, where rapid and precise segmentation is essential.

In this study, we developed a pipeline for segmenting GTVp and GTVn in both pre- and mid-RT T2w MR images. Our findings demonstrate that applying simple preprocessing steps to the acquired MR images can significantly improve the performance of segmentation models. Furthermore, the effective integration of pre-RT data as prior knowledge into mid-RT segmentation models can substantially enhance segmentation performance and model robustness.

Specifically, maximal cropping of image data, elimination of background regions, intensity normalization, and bias-field correction improved segmentation accuracy by an average of nearly 3% for pre-RT and 2% for mid-RT tasks. More importantly, our quantitative metrics reveal that directly integrating pre-RT MR volumes or segmentation masks into the mid-RT pipeline does not lead to meaningful improvements. This is likely because tumor response to treatment can alter tumor appearance, resulting in differing characteristics (even significant shrinkage) in mid-RT volumes. Therefore, directly adding first-time point data, where tumors appear intact, can confuse learning algorithms attempting to map the same segmentation masks onto two different tumor appearances. Conversely, representing the first-time point tumor appearance as probabilistic maps introduces a set of non-binary hypotheses to the segmentation model, resulting in significant accuracy enhancement. In practice, this strategy improved the Dice metric of GTVp by 2% and GTVn by up to 11%.

It is worth to note that we anticipated some performance drops in the testing phase due to the simplification of submitted models necessitated by limited computational resources. Nevertheless, the achieved results remain robust enough to validate the potential of the proposed pipeline.

Finally, while our pipeline yielded promising results for both tasks, certain limitations will be addressed in future studies. In particular, we explored integrating prior information into the segmentation network by simply adding distance maps as second input channels. Future investigations will consider alternative strategies, such as late fusion techniques.

## Figures and Tables

**Table 1.** Quantified conventional volumetric Dice metric for pre-RT task on the training set. The model with overall better performance is marked as bold.

Model	Dice Metric (*μ* ± *σ*)			
	Original Data		Preprocessed Data	
	GTVp	GTVn	GTVp	GTVn
SegResNet	0.638 ± 0.060	0.718 ± 0.057	0.642 ± 0.053	0.740 ± 0.048
ResENC	0.627 ± 0.088	0.723 ± 0.062	0.680 ± 0.079	0.755 ± 0.053
MedNeXt	0.639 ± 0.073	0.754 ± 0.051	0.657 ± 0.083	0.785 ± 0.048
U-Mamba	0.619 ± 0.081	0.726 ± 0.059	0.643 ± 0.087	0.739 ± 0.067
**Ensemble** **(ResENC&MedNeXT)**	--	--	**0.680** ± **0.079**	**0.785** ± **0.048**

**Table 2.** Quantified conventional volumetric Dice metric of nnU-Net ResENC model for mid-RT task on the training set with different combinations of prior information. The model with overall better performance is marked as bold.

Data	Dice Metric (*μ* ± *σ*)			
	Original Data		Preprocessed Data	
	GTVp	GTVn	GTVp	GTVn
no-prior	0.459 ± 0.021	0.695 ± 0.026	0.470 ± 0.016	0.709 ± 0.023
preMR-prior	0.454 ± 0.069	0.751 ± 0.026	0.471 ± 0.078	0.763 ± 0.019
preMask-prior	0.439 ± 0.063	0.750 ± 0.025	0.459 ± 0.068	0.772 ± 0.023
preMRMask-prior	0.457 ± 0.072	0.766 ± 0.018	0.468 ± 0.063	0.778 ± 0.015
**preDistance-prior**	**0.473** ± **0.053**	**0.796** ± **0.013**	**0.493** ± **0.054**	**0.810** ± **0.008**

**Table 3.** Performance of submitted containerized algorithms on testing datasets in terms of aggregated Dice metric.

Testing phase	Aggregated Dice Metric			
	pre-RT		mid-RT	
	GTVp	GTVn	GTVp	GTVn
Preliminary development (n = 2)	0.868	0.929	0.746	0.814
Final (n = 50)	0.795	0.849	0.553	0.865
